# Relationship between Body Composition and Biochemical Parameters with Antioxidant Status in a Healthy Cohort of Postmenopausal Women

**DOI:** 10.3390/metabo12080746

**Published:** 2022-08-14

**Authors:** Héctor Vázquez-Lorente, Lourdes Herrera-Quintana, Jorge Molina-López, Yenifer Gamarra-Morales, Beatriz López-González, Elena Planells

**Affiliations:** 1 Institute of Nutrition and Food Technology “José Mataix”, University of Granada, 18071 Granada, Spain; 2 Faculty of Education, Psychology and Sports Sciences, University of Huelva, Avd. De las Fuerzas Armadas S/N, 21007 Huelva, Spain

**Keywords:** total antioxidant capacity, superoxide dismutase, glutathione peroxidase, body composition, postmenopausal women, menopause

## Abstract

An adequate prooxidant–antioxidant balance—which may be influenced by body composition and biochemical status—is essential to maintain human health, especially in circumstances under which the antioxidant defense decreases, such as menopause. The present study aimed to examine the relationship between body composition and biochemical parameters with antioxidant status in a healthy cohort of postmenopausal women. This cross-sectional study was carried out in a cohort of 78 postmenopausal women aged 44–76 years. The body composition profile was assessed through bioelectrical impedance. The determination of the total antioxidant capacity and superoxide dismutase activity was conducted by the colorimetric method, and glutathione peroxidase activity was determined by the enzymatic immunological method. The vitamin D levels were measured by ultra-performance liquid chromatography–tandem mass spectrometry. The mineral status was assessed through flame atomic absorption spectrophotometry. The rest of the biochemical parameters were assessed through an immunoassay. The total antioxidant capacity and antioxidant gap were negatively influenced by body composition (all *p* ≤ 0.049) and positively related to protein metabolism parameters (all *p* ≤ 0.048), whereas circulating levels of different micronutrients (all *p* ≤ 0.048) and enzymes (all *p* ≤ 0.047) appeared to play an important role in the glutathione peroxidase and superoxide dismutase activities. In conclusion, the menopause-related antioxidant status changes may be influenced by key body composition and biochemical profiles. To confirm this statement, further trials aiming to evaluate the body composition and biochemical intervention-induced changes upon antioxidant defense are needed.

## 1. Introduction

Menopause occurs progressively between the ages of 45 and 55 years during the life of women, and is characterized by permanent loss of menstrual cycles and a significant decrease in the estrogen and progesterone levels, influencing many organs, systems, and processes [1]. The estrogen levels are closely associated with the circulating redox status, thus controlling, in part, circulating antioxidant levels [2]. In this regard, estrogen’s capacities to prevent oxidative stress processes and to produce antioxidant molecules decrease in menopause [3]. Furthermore, the mechanisms involved in antioxidant protection deteriorate with age [4], with an adequate prooxidant–antioxidant balance being essential to maintaining healthy conditions for physiological activities [5].

The total antioxidant capacity (TAC) gathers the synergic and redox interactions between the different antioxidant molecules present in foods and biological fluids [6]. The TAC has been observed to be reduced in postmenopausal women compared with premenopausal women [7]. In order to assess and understand the antioxidant status, antioxidant enzymes, such as superoxide dismutase (SOD) and glutathione peroxidase (GPx), need to be evaluated [8]. SOD faces oxidative stress by catalyzing the dismutation of superoxide into oxygen and hydrogen peroxide [9], whereas GPx is involved in preventing the harmful accumulation of intracellular hydrogen peroxide, thus reducing oxidative stress [10]. In this regard, SOD has been suggested to be one of the most significant antioxidants in the human body, and its status may be compromised in menopausal stages [11]. Moreover, the deprivation of estrogens decreases the gene expression of GPx, thus decreasing its circulating levels [12]. Altogether, the SOD and GPx enzyme activities in the ovaries of postmenopausal women have been observed to be significantly lower than those in the premenopausal stage [13]. Regretfully, there is a lack of information available on the association of plasma TAC with individual endogenous and exogenous antioxidant components in humans, especially in postmenopausal women [8]. Changes in body composition, with gains in fat mass (FM) and losses of fat-free mass (FFM), are menopausal transition-related processes [14]. The previously mentioned changes in the body composition, together with anthropometrical disturbances, have been shown to decrease antioxidant defense in menopausal women [15]. In this regard, although the TAC levels and SOD and GPx activities may be influenced by several factors (e.g., aging, body mass index (BMI), and gender), evidence in this field to date has been conflicting [16].

Therefore, research on the relationship between the antioxidant status and menopausal status has not yet been completely elucidated [17]. In this regard, knowledge of the mechanisms based on the biochemical and body composition parameters that could influence antioxidant defense is needed, and could help to understand the behavior of antioxidant defense in humans at risk of antioxidant disturbances (e.g., postmenopausal women) [11]. Therefore, the present study aimed to examine the relationship between body composition and biochemical parameters with antioxidant status in a healthy cohort of postmenopausal women.

## 2. Materials and Methods

### 2.1. Study Design and Participants

A cohort of 78 healthy postmenopausal women volunteers from the province of Granada, Spain, aged 44–76 years was included in the present cross-sectional study. All individuals signed written informed consent. The study was performed following the principles of the Declaration of Helsinki [18] and approved by the Ethics Committee of the University of Granada (149/CEIH/2016), in accordance with the International Conference on Harmonization/Good Clinical Practice Standards. The inclusion criteria were: (i) presenting postmenopausal status (with at least 12 months of amenorrhea), (ii) accepting participation in the study after being informed about it, and (iii) presenting normal parameters from a previous routine hospital laboratory analysis. The exclusion criteria were: (i) refusing to participate in the study, (ii) taking vitamin and mineral supplements, (iii) presenting any pathology that could affect their nutritional status (i.e., the main components of metabolic syndrome, celiac disease, bulimia, and anorexia), (iv) undergoing hormone-replacement therapy, and (v) presenting systemic inflammatory status (C-reactive protein (CRP) was included as a reference biomarker to assess the inflammation status of the participants)—the cutoff value being below 5.00 mg/L.

### 2.2. Sociodemographic Data Collection

Blood pressure (BP) classified as normal pressure/high pressure was measured three times at 30 s intervals in seated participants with an electronic sphygmomanometer (HBP-9020, OMRON Co. Ltd., Kyoto, Japan) after the participants had rested for 10 min, and the mean value was used for analysis. BP values above 120 mmHg/80 mmHg, for systolic and diastolic pressure, respectively, were considered high pressure values. The following data were obtained through manual questionnaires administered by the researcher: (i) Physical exercise was defined as sedentary/non-sedentary, classifying as non-sedentary a woman who reported less than 30 min/day of regular exercise, less than 3 days/week. (ii) Smoking habits were classified as non-smoker/smoker; a smoker was the subject who smoked more than 1 cigarette/day. (iii) Educational level was divided into basic educational level/secondary or high educational level; individuals presenting the basic educational level were those who only finished primary studies.

### 2.3. Body Composition and Anthropometry Analysis

Height (m) was determined using a stadiometer (Seca, model 213, range 85 to 200 cm; precision: 1 mm; Hamburg, Germany). The individuals’ body weight (kg), FM (kg and %), and FFM (%) were obtained through bioelectrical impedance (Tanita MC-980 Body Composition Analyzer MA Multifrequency Segmental, Barcelona, Spain). The analyzer complies with the applicable European Standards (93/42EEC, 90/384EEC) for use in the medical industry. The subjects of the study were informed in advance of the required conditions prior to measurement: (i) no alcohol intake in the previous 24 h, (ii) no vigorous exercise in the previous 12 h, (iii) no food or drink intake in the previous 3 h, and (iv) no urination immediately before measurement. The BMI was calculated as weight (kg)/height (m^2^). The waist perimeter (WP) was measured at the midpoint between the lower margin of the least palpable rib and the top of the iliac crest [19]. The hip perimeter (HP) was determined with the tape parallel to the floor, at the widest portion of the buttocks [20]. The waist/hip ratio was calculated as the waist perimeter divided by the hip perimeter.

### 2.4. Samples Treatment and Analysis

All samples were obtained in the morning under fasting conditions through blood extraction in the antecubital vein. Plasma was separated by centrifugation at 3000 rpm for 4 min at 4 °C, and the aliquots were frozen at −80 °C until analysis.

The circulating glucose, creatinine, urea, uric acid, total bilirubin, total proteins, albumin, prealbumin, transferrin, CRP, homocysteine (Hcy) glutamic oxaloacetic transaminase (GOT), glutamic pyruvic transaminase (GPT), gamma-glutamyl transferase (GGT), amylase, lactate dehydrogenase (LDH), triglycerides (TG), high-density lipoprotein (HDL), low-density lipoprotein (LDL), total cholesterol (TC), osteocalcin, parathyroid hormone (PTH), and leptin levels were analyzed in the Analysis Unit—which provided reference values—at the Virgen de las Nieves Hospital, Granada (ECLIA, Elecsys 2010 and Modular Analytics E170, Roche Diagnostics, Mannheim, Germany). The plasma calcium (Ca), magnesium (Mg), iron (Fe), and copper (Cu) levels were obtained through flame atomic absorption spectrophotometry (FAAS, Perkin Elmer^®^ Analyst 300 model, Berlin, Germany), and the plasma phosphorous (P) levels were determined through the Fiske–Subbarow colorimetric method (Thermo Scientific, Rockford, IL, United States). Folic acid (Fol) and vitamin B_12_ (B_12_) were measured using a DxI^®^ Autoanalyzer (Beckman Coulter, CA, USA) employing a competitive electrochemiluminescence immunoassay for quantitative determinations. Vitamin D_3_ and vitamin D_2_ were measured by liquid chromatography–tandem mass spectrometry (LC-MS/MS) using a Waters Acquity UHPLC I-Class System Chromatograph (Waters, London, UK). Total vitamin D was calculated as vitamin D_3_ + vitamin D_2_. TAC determination was conducted by evaluating the reduction power of Cu^2+^ from the action of antioxidants present in samples (TAC kit, Jaica, Shizuoka, Japan). The GPx activity was determined by the enzymatic immunological method using the Bioxytech GPx-340™ kit (OxisResearch™, Portland, OR, USA). The SOD activity was determined by the colorimetric method based on cytochrome c reduction using a Randox Ransod kit (RANDOX Laboratories Ltd., Crumlin, UK). In addition, we calculated the SOD/GPx ratio and antioxidant gap (GAP) with the Equation [21]:

$$\mathrm{GAP}\;\left(\frac{µ\mathrm{mol}}{L}\right)=\mathrm{TAC}\;\left(\frac{µ\mathrm{mol}}{L}\right)-\left[\left(\mathrm{albumin}\;\left(\frac{µ\mathrm{mol}}{L}\right)\;\times \;0.69\right)+\mathrm{uric}\;\mathrm{acid}\;\left(\frac{µ\mathrm{mol}}{L}\right)\right]$$

The remainder of the above-mentioned parameters were analyzed in the Scientific Instrumentation Center (SIC) at the University of Granada, where reference values were provided. All biochemical parameters were analyzed in the plasma. All samples were measured in one run, in duplicate, in the same assay batch, and blinded quality control samples were included in the assay batches.

### 2.5. Statistical Analysis

Data were obtained using SPSS 22.0 Software for MAC (SPSS Inc. Chicago, IL, USA). GraphPad Prism 9 software (GraphPad Software, San Diego, CA, USA) was used for plotting the graphs. As a previous step to the execution of a parametric model or not, the hypothesis of normal distribution was accepted using the Kolmogorov–Smirnov test. Categorical variables are shown as frequencies (N) and percentages (%). Continuous variables are presented as the mean and standard deviation (X ± SD). We performed sample size calculation for our primary aim of the cross-sectional study based on the relationship between the body composition and biochemical parameters with antioxidant status in a healthy cohort of postmenopausal women by using G*Power software (version 3.1.9.6, Kiel, Germany). The number of participants to be included in the study was calculated based on the main statistical method used (unpaired t-test). An a priori power analysis indicated that a total of at least 70 participants were required. This calculation was based on a moderate effect size (effect size d = 0.70), an alpha level of 0.05, and a beta value of 0.90 for an unpaired t-test calculating the difference between two independent means (two groups) [22]. Student’s unpaired-samples test was conducted to study differences between early and late menopause. Pearson’s correlation models were used to evaluate the significant associations of body composition and biochemical parameters with TAC, GPx, and SOD. Simple linear regression models were used to evaluate the significant associations of the body composition and biochemical parameters with the SOD/GPx ratio and GAP, (Model 0) and adjusted by age, BMI, physical activity, and early vs. late menopause (Model 1). β (standardized regression coefficient), R^2^, and *p* values from simple linear regression analyses were obtained. A *p*-value of less than 0.05 was considered significant.

## 3. Results

One in four participants had a sedentary status, and almost half presented high BP. Moreover, a fifth of the subjects smoked. Finally, a third of the participants presented a basic educational level (Supplementary Table S1).

Table 1 shows the body composition and biochemical variables of the study. The body composition variables that showed the lowest percentages below the reference values were FM in % (almost the whole population) and waist/hip ratio (one in ten subjects). Regarding the biochemical variables, 25–OH–D (with four in five participants) was the biochemical variable that showed the highest percentage of participants below the reference values. The height, FFM, and albumin were higher in early vs. late menopause (all *p* ≤ 0.048), whereas glucose, urea, uric acid, total bilirubin, and osteocalcin were lower in early vs. late menopause (all *p* ≤ 0.017).

Table 2 shows the antioxidant status variables of the study. TAC and GPx—with half of the subjects—were the biochemical variables that showed the highest percentage of participants below the reference values. All antioxidant parameters showed no significant differences between groups (all *p* > 0.05).

**Figure 1:**
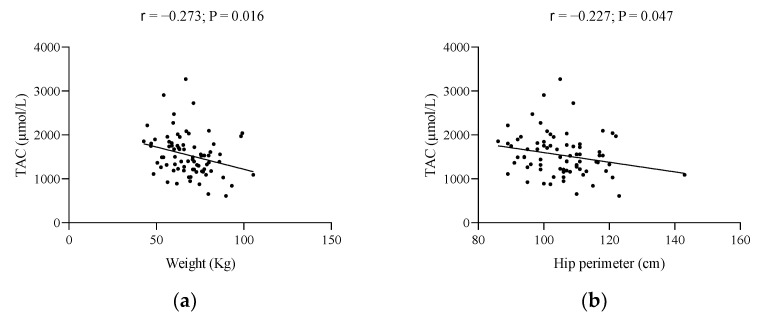

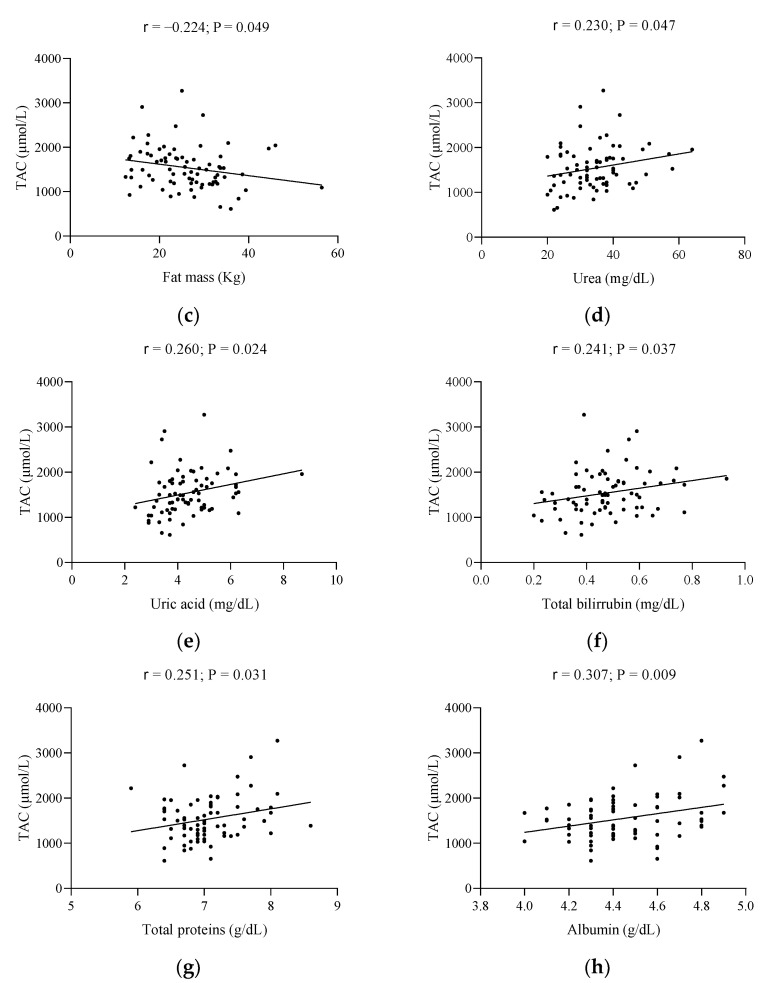
Significant associations of the body composition and biochemical parameters of the study with TAC. (**a**) weight and TAC, (**b**) hip perimeter and TAC, (**c**) fat mass and TAC, (**d**) urea and TAC, (**e**) uric acid and TAC, (**f**) total bilirubin and TAC, (**g**) total proteins and TAC, and (**h**) albumin and TAC. r, Pearson’s correlation coefficient. Abbreviations: TAC, total antioxidant capacity. A *p*-value less than 0.05 was considered significant.

Figure 1 shows the significant associations of the body composition and biochemical parameters of the study with TAC. Regarding body composition, the weight, hip perimeter, and FM expressed in kg showed a significant indirect association with TAC (all *p* ≤ 0.049; Figure 1a–c), whereas BMI showed no significant relationship with TAC (*p* = 0.062) (data not shown). In the case of biochemical parameters, urea, uric acid, total bilirubin, total proteins, and albumin, were seen to be directly related to TAC (all *p* ≤ 0.047; Figure 1d–h).

**Figure 2:**
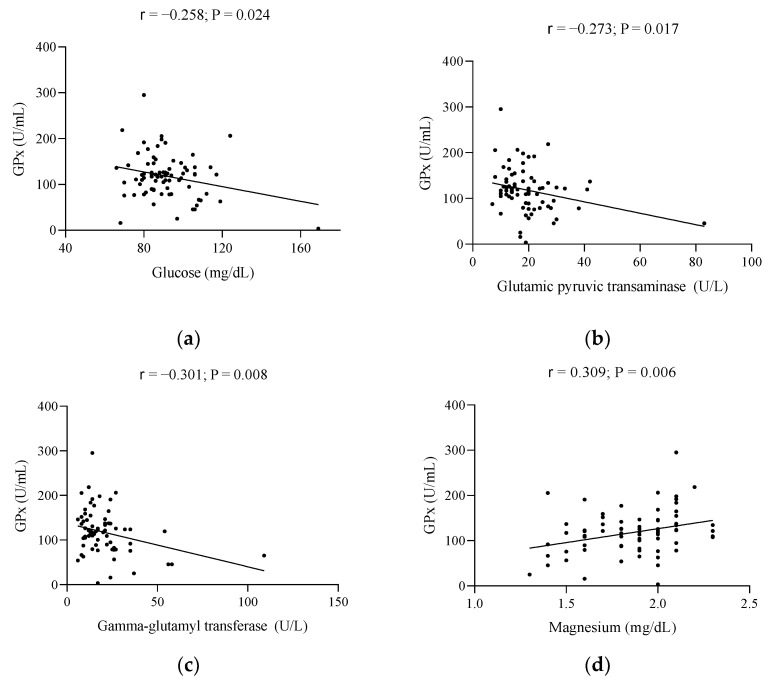
Significant associations of the body composition and biochemical parameters of the study with GPx. (**a**) glucose and GPx, (**b**) glutamic pyruvic transaminase and GPx, (**c**) gamma-glutamyl transferase and GPx, and (**d**) magnesium and GPx. r, Pearson’s correlation coefficient. A *p*-value less than 0.05 was considered significant. Abbreviations: GPx, glutathione peroxidase; GPT, glutamic pyruvic transaminase; GGT, gamma-glutamyl transferase; Mg, magnesium.

Figure 2 represents the significant relationships of the body composition and biochemical parameters of the study with GPx. Regarding body composition, no significant associations (data not shown) were observed with GPx (all *p* > 0.05). When referring to biochemical parameters, glucose, GPT, and GGT were seen to be inversely associated with GPx (all *p* ≤ 0.024; Figure 2a–c), whereas a direct association of Mg with GPx was observed (*p* ≤ 0.006, Figure 2d).

**Figure 3:**
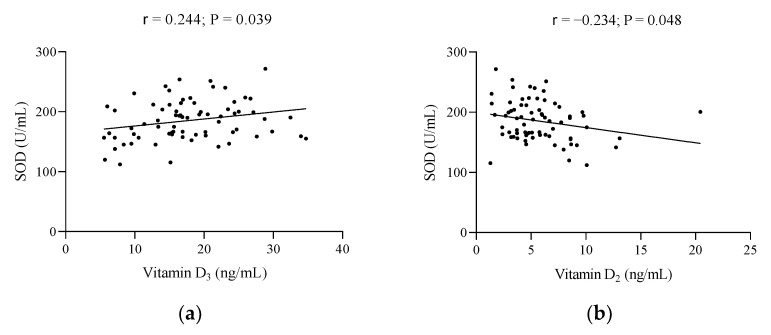

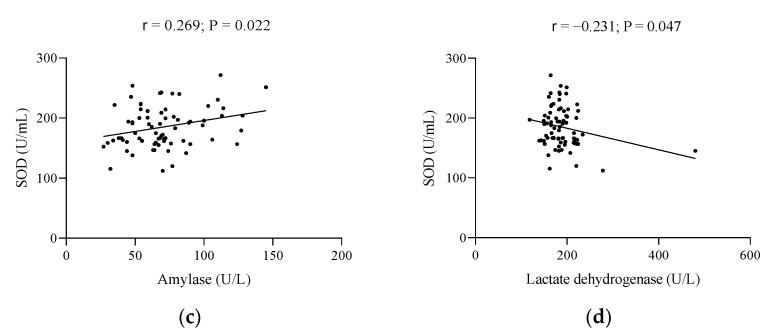
Significant associations of the body composition and biochemical parameters of the study with SOD. (**a**) Vitamin D_3_ and SOD, (**b**) Vitamin D_2_ and SOD, (**c**) amylase and SOD, and (**d**) lactate dehydrogenase and SOD. r, Pearson’s correlation coefficient. A *p*-value less than 0.05 was considered significant. Abbreviations: SOD, superoxide dismutase.

Figure 3 shows the significant associations of the body composition and biochemical parameters of the study with SOD. No significant associations (data not shown) were observed (all *p* > 0.05) for the body composition variables and SOD activity. In the case of biochemical parameters, 25-OH-D_3_ and amylase were seen to be directly related to SOD (all *p* ≤ 0.039, Figure 3a,c), whereas an inverse association was seen between 25-OH-D_2_ and LDH (all *p* ≤ 0.048, Figure 3b,d).

Table 3 shows the results of regression analysis between the body composition and biochemical parameters of the study and the SOD/GPx ratio and GAP. No significant associations (data not shown) were observed (all *p* > 0.05) between the SOD/GPx ratio and body composition variables. In the case of the biochemical parameters, the SOD/GPx ratio showed a direct relationship with the circulating glucose levels, which persisted after adjusting by confounders (all *p* ≤ 0.001, Model 0 and Model 1). Regarding the relationship between GAP and the body composition, GAP showed an inverse association with weight, HP, FM, and FFM (all *p* ≤ 0.021, Model 0), which lost their significance after adjusting by covariates (all *p* > 0.078, Model 1). Additionally, GAP was negatively associated with BMI, which persisted after adjusting by confounders (all *p* ≤ 0.020, Model 0 and Model 1). In the case of the biochemical variables of the study, GAP was directly related to albumin, which persisted after including covariates (all *p* ≤ 0.034, Model 0 and Model 1). Moreover, GAP, which was directly related to the total proteins (*p* = 0.048, Model 0), lost its significance after including covariates (*p* = 0.095, Model 1).

## 4. Discussion

The main results of the present study show that the activity of TAC and GAP were influenced by body composition, along with protein metabolism parameters. Moreover, the circulating levels of different micronutrients and enzymes may play an important role in GPx and SOD. These findings shed light on the idea that both the body composition and biochemical parameters are decisive for maintaining an adequate antioxidant status in postmenopausal women.

Various metabolic factors may influence TAC, which tends to increase at an early stage of overweightness and obesity, possibly as a compensatory response to oxidative stress [23]. However, TAC is usually significantly lower in obese subjects compared with non-obese subjects [15,24]. Along this line, our results showed a significant inverse association of weight, HP, and FM (expressed in kg) with TAC, whereas no such significant relationships were observed when considering BMI. In the literature, the WP and HP, weight, BMI, and body fat percentage, among others, have been negatively correlated with TAC [15,25]. On the other hand, urea, uric acid, total bilirubin, total proteins, and albumin were seen to be directly related to TAC. The main determinants of plasma TAC are albumin, bilirubin, and uric acid [26], and positive correlations have been widely reported between them in the literature [27,28,29,30]. In fact, it has been suggested that uric acid plays a specific role in the antioxidant response due to the different kinetics of TAC on the plasma and serum uric acid levels [31]. Furthermore, the antioxidant activity of uric acid has been estimated to account for approximately 50–65% of the TAC of biological fluids in humans [32,33]. These relationships have been studied in different pathologies, such as renal disease, with high TAC probably caused by the accumulation of urea in serum [34], as well as a decrease in plasma TAC after dialysis, which was equivalent to a diminution in creatinine, urea, and uric acid levels [35].

Regarding GPx activity, it was positively influenced by Mg. This relationship has been reported previously in subjects with diabetes, supporting the hypothesis that hypomagnesemia may influence the development of oxidative stress in these subjects [36]. Moreover, the glucose, GPT, and GGT levels were inversely related to GPx activity. In diabetic patients, fasting glucose has been negatively correlated with GPx, which may be due to the role of hyperglycemia upon increasing oxidative stress through several pathways [37]. Likewise, fasting serum glucose has been correlated with GGT, with higher GGT levels and lower GPx activity in patients with type 2 diabetes compared with a control group [38]. On the other hand, the elevation of GPT has been associated with significant changes in GPx activity in animal models [39], and the use of a methanolic extract has shown a preventive effect on the increase in GPT, enhancing GPx activity [40].

The results of SOD activity showed negative influences of vitamin D_2_ and LDH, and positive influences of vitamin D_3_ and amylase. Vitamin D_3_ has been demonstrated to diminish the formation of free radicals by enhancing antioxidative defense systems, including SOD [41]. Meanwhile, an enriched diet combined with vitamin D_2_ appears to increase SOD in animal models [42]. However, the role of vitamin D is not clear—its supplementation did not produce significant changes in erythrocyte SOD in patients with hearing loss – [43]. With regard to LDH, increased SOD levels along with decreased LDH levels, and vice versa, have been observed in critical patients, with lower SOD levels and abnormal activity of LDH being a possible indicator of worse prognosis or infection severity [44]. The same tendency has been observed after bariatric surgeries, with increased amylase and SOD levels, and decreased LDH [45]. However, in diabetic patients, amylase activity was increased, whereas SOD activity was decreased [46]. Moreover, higher blood amylase levels have been positively related to higher insulin sensitivity in pig models after bariatric surgery [47]. All of this suggests that any disturbance in biochemical metabolism may increase oxidative stress, especially those parameters related to glycemia.

SOD and GPx are important antioxidant enzymes in humans—SOD converts superoxide anion radicals into oxygen and hydrogen peroxide, which is then converted by GPx to water and oxygen—and the SOD/GPx ratio is more relevant than the absolute activities of the individual enzymes [48]. Indeed, it has been suggested that an imbalance in this ratio results in the accumulation of hydrogen peroxide, and may be an important factor of cellular aging [49]. In our cohort, the SOD/GPx ratio was directly associated with glucose, which is probably due to the observed negative correlation between the glucose levels and GPx activity.

Finally, the total proteins and albumin were directly associated, and weight, BMI, HP, FM, and FFM were inversely related with GAP. These associations persisted after adjusting by confounders (i.e., age, BMI, physical activity, and early vs. late menopause). The principal antioxidants (by mass and activity) of human plasma are albumin and uric acid, with GAP being a reflection of the combined activity of other extracellular antioxidants [50]. In our study, the main determinant of GAP appears to be weight, since both FM and FFM would have similar effects on GAP. However, more evidence is needed to understand the role of body composition in antioxidant defense.

The present study suffers from some limitations, including: (i) its cross-sectional design, which means that no causal relationships can be established, (ii) the lack of dietary assessment, which could have helped to enrich the study, (iii) the lack of information about other minor antioxidant parameters, which could have helped to enrich the way to interpret and confirm our results, (iv) fewer participants than desired being recruited, and (v) the study population being limited to postmenopausal women aged from 44–76 years old from a specific area of southern Spain; hence these results may not be generalizable to postmenopausal women of different regions or with ages not included in our range.

## 5. Conclusions

In conclusion, the menopause-related antioxidant status changes may be influenced by body composition and biochemical profile. To confirm this statement, further trials aimed at evaluating body composition and biochemical intervention-induced changes upon antioxidant defense are needed.

## Figures and Tables

**Table 1:** Body composition and biochemical variables of the study.

Characteristics	Total Population (*n* = 78)	Below Reference	Early Menopause (*n* = 39)	Late Menopause (*n* = 39)	Reference Values
	Mean ± SD	%	Mean ± SD	Mean ± SD	Range
**Body Composition**					
Weight (kg)	68.7 ± 13.2	-	70.3 ± 13.9	66.9 ± 12.3	-
Height (m)	159.3 ± 6.23	-	161.5 ± 6.20	156.8 ± 5.30 *	-
BMI (kg/m^2^)	27.0 ± 4.60	14.1	26.9 ± 4.80	27.2 ± 4.40	22.0–27.0
WP (cm)	89.0 ± 12.6	50.0	87.8 ± 12.4	90.5 ± 13.0	< 90.0
HP (cm)	105.8 ± 10.5	65.4	105.7 ± 9.50	105.9 ± 11.6	< 110.0
Waist/hip ratio	0.83 ± 0.08	12.8	0.80 ± 0.10	0.80 ± 0.10	< 0.80
FM (%)	37.6 ± 5.92	1.30	37.4 ± 5.50	37.8 ± 6.40	23.0–31.0
FM (kg)	26.3 ± 8.48	-	26.8 ± 8.50	25.7 ± 8.60	-
FFM (%)	62.4 ± 5.92	100.0	43.5 ± 6.30	40.2 ± 8.30 *	> 69.0
**Biochemical parameters**					
Glucose (mg/dL)	92.2 ± 15.9	3.90	87.4 ± 12.4	97.4 ± 17.9 *	70.0–110.0
Creatinine (mg/dL)	0.69 ± 0.13	2.60	0.67 ± 0.10	0.73 ± 0.20	0.50–0.90
Urea (mg/dL)	34.5 ± 9.08	0.00	32.2 ± 8.00	37.2 ± 9.60 *	10.0–50.0
Uric acid (mg/dL)	4.40 ± 1.07	0.00	4.10 ± 0.90	4.70 ± 1.20 *	2.40–5.70
Total bilirubin (mg/dL)	0.47 ± 0.14	0.00	0.40 ± 0.10	0.50 ± 0.10 *	0.10–1.20
Total proteins (g/dL)	7.08 ± 0.52	14.7	7.20 ± 0.50	7.00 ± 0.50	6.60–8.70
Albumin (g/dL)	4.44 ± 0.21	0.00	4.50 ± 0.20	4.40 ± 0.20 *	3.50–5.20
Prealbumin (mg/dL)	25.2 ± 5.07	11.1	25.6 ± 4.50	24.6 ± 5.80	20.0–40.0
Transferrin (mg/dL)	280.2 ± 45.9	3.20	279.0 ± 43.1	281.8 ± 50.0	200.0–360.0
CRP (mg/L)	1.04 ± 6.95	0.00	1.70 ± 9.30	0.20 ± 0.20	0.02–5.00
Hcy (µmol/L)	11.7 ± 4.76	73.3	11.6 ± 4.45	11.8 ± 5.17	< 13.0
GOT (U/L)	22.3 ± 6.47	97.4	22.1 ± 4.80	22.5 ± 8.00	< 37.0
GPT (U/L)	19.7 ± 10.5	96.1	19.0 ± 7.20	20.6 ± 13.4	< 41.0
GGT (U/L)	20.0 ± 14.8	19.7	19.9 ± 17.3	20.1 ± 11.5	11.0–50.0
Amylase (U/L)	69.8 ± 25.5	9.50	66.0 ± 23.6	74.1 ± 27.1	40.0–140.0
LDH (U/L)	186.4 ± 46.3	1.30	183.9 ± 53.2	189.3 ± 37.3	110.0–295.0
TG (mg/dL)	108.2 ± 67.9	3.90	108.2 ± 82.0	108.2 ± 48.4	50.0–200.0
HDL (mg/dL)	66.6 ± 15.6	1.30	66.9 ± 12.1	66.4 ± 19.0	40.0–60.0
LDL (mg/dL)	128.0 ± 31.3	3.90	126.4 ± 30.3	130.0 ± 32.8	70.0–190.0
TC (mg/dL)	220.5 ± 34.4	0.00	219.1 ± 33.7	222.1 ± 35.6	110.0–200.0
Osteocalcin (ng/mL)	15.3 ± 9.82	48.0	12.8 ± 8.60	18.3 ± 10.5 *	15.0–46.0
PTH (pg/mL)	56.2 ± 23.8	0.00	54.9 ± 27.0	57.8 ± 19.4	20.0–70.0
Leptin (ng/mL)	13.9 ± 4.83	0.00	14.0 ± 5.10	13.8 ± 4.6	3.60–11.1
Folic acid (ng/mL)	11.2 ± 4.09	0.00	10.8 ± 4.29	11.6 ± 3.85	2.70–17.0
Vitamin B_12_ (pg/mL)	527.1 ± 271.9	1.40	527.5 ± 220.9	526.5 ± 330.7	190.0–900.0
25–OH–D (ng/mL)	23.5 ± 7.40	79.2	23.7 ± 7.80	23.3 ± 7.10	30.0–100.0
25–OH–D_3_ (ng/mL)	17.7 ± 7.06	62.5	17.7 ± 7.10	17.8 ± 7.10	> 20
25–OH–D_2_ (ng/mL)	5.74 ± 3.11	93.1	6.00 ± 3.30	5.50 ± 2.90	> 10
Ca (mg/dL)	9.21 ± 0.44	6.50	9.20 ± 0.40	9.30 ± 0.50	8.60–10.2
P (mg/dL)	3.49 ± 0.50	3.90	3.40 ± 0.50	3.60 ± 0.40	2.70–4.50
Mg (mg/dL)	1.87 ± 0.25	23.1	1.90 ± 0.20	1.90 ± 0.30	1.70–2.20
Fe (µg/dL)	92.6 ± 30.7	13.0	88.3 ± 32.8	97.5 ± 27.8	60.0–170.0
Cu (µg/dL)	101.4 ± 23.0	27.0	107.3 ± 23.0	92.9 ± 20.8 *	85.0–180.0

*n* = 78. All variables are expressed as the mean ± standard deviation (SD). % represents the percentage of subjects below the reference values. Early and late menopause women are those below and above the median age (i.e., 57 years), respectively. T-Student’s unpaired-samples test was used for comparing the mean differences between early and late menopause. * significant mean differences with *p*-values less than 0.05. Abbreviations: BMI, body mass index; B_12_, vitamin B_12_; Ca, calcium; Cu, copper; CRP, C-reactive protein; Fe, iron; FFM, fat-free mass; FM, fat mass; Fol, folic acid; GGT, gamma–glutamyl transferase; GOT, glutamic oxaloacetic transaminase; GPT, glutamic pyruvic transaminase; Hcy, homocysteine; HDL, high-density lipoprotein; HP, hip perimeter; LDH, lactate dehydrogenase; LDL, low-density lipoprotein; Mg, magnesium; P, phosphorous; PTH, parathyroid hormone; TC, total cholesterol; TG, triglycerides; WP, waist perimeter.

**Table 2:** Antioxidant status variables of the study.

Characteristics	Total Population (*n* = 78)	Below Reference	Early Menopause (*n* = 39)	Late Menopause (*n* = 39)	Reference Values
	Mean ± SD	%	Mean ± SD	Mean ± SD	Range
**Antioxidant Parameters**					
TAC (µmol/L)	1539.3 ± 483.1	51.9	1534 ± 594.8	1545.7 ± 308.1	1500.0
GPX (U/mL)	118.2 ± 47.7	50.6	117.3 ± 40.9	119.4 ± 55.4	120.0
SOD (U/mL)	184.4 ± 34.2	31.2	184.5 ± 34.5	184.2 ± 34.4	164.0–240.0
SOD/GPx ratio	2.42 ± 4.97	0.00	1.90 ± 1.45	3.03 ± 7.20	–
GAP (µmol/L)	823.9 ± 473.3	–	845.6 ± 593.2	799.8 ± 293.9	–

*n* = 78. All variables are expressed as the mean ± standard deviation (SD). % represents the percentage of subjects below the reference values. Early and late menopause women are those below and above median age (i.e., 57 years), respectively. T-Student’s unpaired-samples test was used for comparing the mean differences between early and late menopause. Significant was set for *p*-values less than 0.05. Abbreviations: GPx, glutathione peroxidase; TAC, total antioxidant capacity; SOD, superoxide dismutase.

**Table 3:** Regression analysis between the body composition and biochemical parameters of the study and the SOD/GPx ratio and GAP.

Characteristics	Model 0			Model 1		
	ß	R^2^	P	ß	R^2^	P
**SOD/GPx Ratio**						
Glucose (mg/dL)	0.425	0.309	**0.001**	0.582	0.312	**0.001**
**GAP**						
Weight (kg)	−0.310	0.096	**0.008**	−0.309	0.096	0.250
BMI (kg/m^2^)	−0.274	0.075	**0.020**	−0.278	0.080	**0.012**
HP	−0.274	0.075	**0.020**	−0.271	0.076	0.854
FM	−0.272	0.075	**0.021**	−0.270	0.075	0.994
FFM	−0.285	0.081	**0.015**	−0.283	0.081	0.078
Total proteins (g/dL)	0.235	0.055	**0.048**	0.246	0.062	0.095
Albumin (g/dL)	0.267	0.071	**0.023**	0.295	0.085	**0.034**

ß, standardized regression coefficient. R^2^ and P are from simple and multiple regression analyses between age and the significant biochemical parameters: Model 0, simple regression analysis; Model 1, multiple regression analysis adjusted by age, BMI, physical activity, and early vs. late menopause. Bold numbers indicate a statistically significant association. Significance was set at *p*-value < 0.05. Abbreviations: BMI, body mass index; GAP, antioxidant GAP; HP, hip perimeter; FM, fat mass; FFM, fat-free mass.

## Data Availability

Not applicable.

## Supplementary Materials

The following supporting information can be downloaded at: https://www.mdpi.com/article/10.3390/metabo12080746/s1, Table S1: Sociodemographic variables of the study.
