# Supplementary material for: Relationship between Body Composition and Biochemical Parameters with Antioxidant Status in a Healthy Cohort of Postmenopausal Women
Source: Metabolites. 2022 Aug 14;12(8):746. doi: 10.3390/metabo12080746 (PMC9412505; doi:10.3390/metabo12080746)
Supplement: Supplementary file 1 [file metabolites-12-00746-s001.zip › metabolites-1840948-supplementary.pdf]

Table S1.- Sociodemographic variables of the study.

Characteristics	Total population (n = 78)	
	**N**	**(%)**
**Sociodemographic**		
**Blood pressure**	-	-
Normal blood pressure	43	(55)
High blood pressure	35	(45)
**Physical exercise**	-	-
Sedentary	20	(26)
Non-sedentary	58	(74)
**Smoking habit**	-	-
Non-smoker	62	(80)
Smoker	16	(20)
**Educational level**	-	-
Basic educational level	29	(37)
Secondary or high educational level	49	(63)

N = 78. All variables are expressed as frequencies (N) and percentages (%).
